# Development and evaluation of mucoadhesive bigel containing tenofovir and maraviroc for HIV prophylaxis

**DOI:** 10.1186/s43094-020-00093-3

**Published:** 2020-11-14

**Authors:** Margaret O. Ilomuanya, Ayotunde T. Hameedat, Edidiong N Akang, Sabdat O. Ekama, Boladale O. Silva, Alani S Akanmu

**Affiliations:** 1 Department of Pharmaceutics and Pharmaceutical Technology, Faculty of Pharmacy, University of Lagos, PMB 12003, Surulere, Lagos, Nigeria; 2 Center for Biomedical Research, Population Council, New York, 10065 USA; 3 Department of Anatomy, College of Medicine, University of Lagos, PMB 12003, Surulere, Lagos, Nigeria; 4 Clinical Sciences Division, Nigerian Institute of Medical Research, 6 Edmund Crescent, P.M.B. 2013 Yaba, Lagos, Nigeria; 5 Department of Hematology and Blood Transfusion, College of Medicine, University of Lagos, Idi-Araba, Lagos State, Nigeria

**Keywords:** Tenofovir, Maraviroc, HIV, Bigel, Microbicides

## Abstract

**Background:**

Sexual transmission of HIV is the most common means of acquiring the disease. Topical microbicides have been investigated to prevent transmission. This study will use a specific entry inhibitor, maraviroc, and a nucleotide reverse transcriptase inhibitor (NRTI), tenofovir, a dual combination which will provide a synergist effect that can enhance the efficacy of HIV microbicides via a mucoadhesive dual compartment bigel. Bigel formulation via hydrogel organogel linkages were developed and evaluated for their physicochemical characteristics, safety, and anti-HIV efficacy. In vitro diffusion studies were performed with Franz diffusion cells having effective diffusion surface area of 1.76cm^2^ and receiver chamber volume of 15mL.

**Result:**

The bigel formulations showed a viscosity ranging from 14179 to 14560 cPs and had a good spreadability and acidic pH in the range of 4.0 ± 0.34 to 5.2 ± 0.18. The bigel formulations showed good anti-HIV activity at a concentration of 0.1 μg/mL. The in vitro release study of maraviroc from the bigel formulations showed a release rate ranging from 2.675 to 3.838 μg/cm^2^/min^½^ while the release rate for tenofovir ranged from 3.475 to 3.825 μg/cm^2^/min^½^. The bigel formulations were non-toxic to the human vagina as there was < 1 log_10_ change in *Lactobacilli crispatus* viability.

**Conclusion:**

This study successfully developed a dual compartment bigel containing maraviroc and tenofovir. BG C was found to be stable and safe towards vaginal and rectal epithelium, and it actively prevented HIV transmission. This bigel has the potential for long-term pre-exposure prophylaxis prevention of HIV transmission.

## Background

Halting the transmission of HIV through prevention to achieve the UNAIDS 95-95-95 goal is one that has been a major focus of research and program interventions over several years now [1]. The female gender is the most vulnerable to getting infected with HIV, with their anatomic constitution predisposing them as a higher risk group compared to the male gender. Thus, it is necessary that any prevention strategy to be developed puts this into consideration [2]. In developed countries, the incidence rate of HIV infection in homosexual couples is comparable to the rate seen among heterosexual couples in sub-Saharan Africa [3]. Transmission of infection in this class of individuals might have been through rectal intercourse, an activity common in men who have sex with men and sometimes heterosexual couples. It is therefore necessary that the focus of HIV microbicides is not restricted to empowering women but also considering these populations.

Pre-exposure prophylaxis through utilization of effective antiretroviral medications has been shown to be effective. Also, the use of highly effective antiretroviral therapies in topical formulations such as antiviral impregnated vaginal rings, tablets, biofilms, and antimicrobial gels has been extensively evaluated [4]. Compounds such as dapivirine, tenofovir, and maraviroc have been evaluated as topical antimicrobial agents in the prevention of HIV infection through sexual exposure, with varying results on efficacy shown among these compounds as topical formulations [4–6]. This might not be unconnected to the class of the antiretrovirals and their mechanism of action, formulation strength and type, and mode and frequency of use, among other factors that might affect the efficacy in both in vitro and in vivo evaluations of these agents [7]. As Africa grapples with the impact of HIV epidemic, it is pertinent to implement strategies to stem the tide of the high prevalence and incidence rates of HIV infection in the region. Antiretroviral therapy can be used to slow the sequence of HIV disease but not stop or cure it. First-line medications combinations such as dolutegravir, tenofovir, and lamivudine are utilized in patients, while protease inhibitors are use as second-line treatments [7]. Topical microbicides provide an effective means of preventing infections in the vaginal and rectal fluid while countering the disadvantages of other pharmacological and physical prevention methods [8]. Women make up about half of the individuals living with the human immunodeficiency virus in sub-Saharan Africa, hence the need to develop female-specific HIV preventive method [9]. The availability of a microbicides with potential for preventing HIV transmission will empower women to control and prevent the transmission of the infection. Furthermore, there is need to extend HIV-preventive method to include rectal formulation that can prevent acquisition of the infection rectally as RAI has been found to be common among both heterosexual and homosexual couples.

Previous work by [10] revealed entry inhibitors deemed as non-specific did not prevent HIV transmission while increasing viral acquisition risk in the participants. However, this study attempts to use a specific entry inhibitor, maraviroc, and a nucleotide reverse transcriptase inhibitor (NRTI), tenofovir, that can inhibit viral replication. The dual combination will provides a synergist effect that can enhance the efficacy of HIV microbicides via a mucoadhesive bigel that will adhere to the walls of the vagina and hence prevent leakage.

## Method

### Materials

The materials used are as follows: refined palm oil, Labrafac (Gattefosse, France); polyoxyethylene sorbitan monooleate (Tween 80 Merck, Germany), Labrafac (propylene glycol dicaprylate (Gattefose, France); trifluoroacetic acid (Macklin Biochemical Ltd. China); hyaluronic acid sodium salt (Merck, Germany); maraviroc analytical standard (Cat# 11580, Lot# 15057WB-67 Fisher Bio-services/NIH-ARP German town MD); maraviroc CAS# 376348-65-1 Shanghai Macklin Biochemical Co., Ltd; nonoxylol (DPT Laboratories Ltd., USA); cellulose membrane (0.45 μm MERCK UK); tenofovir analytical standard (MFCD08141829 Sigma–Aldrich, St. Louis, USA); tenofovir powder (Macklin Biochemical Ltd. China); medroxyprogesterone acetate (Depo Provera®, Pfizer, NY, USA); and Milli-Q-system derived water. All other chemicals and reagents used were used without further processing and were of analytical reagent grade.

### Formulation of the bigel

Maraviroc was dispersed in palm olein and maintained at 60 °C in a water bath with continuous agitation for 10 min. The surfactant mixture of tween 80 (polyoxyethylene sorbitan monooleate) and propylene glycol dicaprylate was introduced to the maraviroc palm olein dispersion (Table 1) and stirred for 30 min using a magnetic stirrer, and water was subsequently added using a pipette until the formation of organogel occurred [11].

**Table 1:** Composition of tenofovir and maraviroc bigel formulations

	Ingredients	Bigel A	Bigel B	Bigel C	Bigel D
Hydrogel	Hyaluronic acid (% w/w)	5	2.5	5	2.5
	Tenofovir (%w/w)	1	1	1	1
Organogel	Tween 80 (% v/w)	38	38	43	43
	Labrafac (% v/w)	19	19	22	22
	Oil (% v/w)	21	21	21	21
	Water (% v/w)	22	22	14	14
	Propylparaben (% w/w)	0.01	0.01	0.01	0.01
	Maraviroc (% w/w)	0.1	0.1	0.1	0.1
**Bigel**	**Organogel to hydrogel ratio**	**1: 1**	**3:2**	**2: 3**	**3:2**

To 1% (w/v) of hyaluronic acid in 100 mL of water, 2.67%w/v sodium periodate solution was titrated at a ratio of 1:1. This solution was retained for 24 h at 25 °C and then dissolved with PBS containing tenofovir (pH 7.4) to 5% and 2.5% hyaluronic acid hydrogel containing tenofovir for the varying hydrogels (Table 1).

Varying bigel formulations were obtained by heating the organogel to 35 °C while introducing it to the hydrogel with continuously stirring. The mixture was eventually cooled to 25 °C. Bigels A, B, C, and D were formulated utilizing varying ratios of hydrogel to organogel as shown in Table 1. Different ratios were utilized to assess the stability of the formulation. All the bigel formulations contained final concentration of 0.1% w/w maraviroc and 1% w/w tenofovir. N9 gel was used as the positive control.

### Physicochemical testing

The pH, spreadability, color, odor, and appearance of the gels were observed at varying time intervals. Using a Brookfield viscometer (Model HADVIII+), viscoelastic properties and rheology of the bigel as a function of time were carried out. Accelerated stability testing was carried out on all formulations to assess the ability of the bigel to maintain structural integrity during varying storage conditions [11, 12].

### Microscopic analysis

The structural features of the bigel formulations were analyzed using optical and scanning electron microscopic techniques developed by Behera et al. [13].

### Compatibility studies

The bigel formulations were scanned via Fourier-transform infrared (FTIR) scanned over 4000–400 cm^−1^ [14]. The nature of the bigel (amorphous or crystalline) and drug-excipient compatibility was elucidated via a differential scanning calorimeter. This was carried out between the temperatures of 0 and 400 °C in nitrogen. The obtained thermograms were observed for any type of interaction [13].

### Mucoadhesion study

Mucin adsorption in the bigel formulations was determined using method of Ilomuanya et al. [11]. Evaluating the absorbance of mucin-bigel-simulated seminal fluid supernatant at 555 nm:

1 $$ \%\mathrm{Total}\ \mathrm{mucin}\ \mathrm{content}\ \mathrm{adsorbed}=\frac{\mathrm{mucin}\ \mathrm{mass}-\mathrm{free}\ \mathrm{mucin}\ \mathrm{mass}}{\mathrm{mucin}\ \mathrm{mass}}\times 100 $$

### In vitro drug release rate

A cellulose membrane containing 1 g of the bigel was clamped between the donor and receiver chambers of the vertical Franz diffusion cells with an available diffusion area of 1.76 cm^2^ at 32 ± 1 °C. At pre-determined time intervals, the concentration of the maraviroc and tenofovir in the sample was determined via a validated high-performance liquid chromatography method [6, 11] and the date was fitted into various kinetic models.

### Safety testing

#### In vitro cytotoxicity

MTT assay using HeLa cells in evaluation of in vitro cytotoxicity was carried using method from Meng et al. [8].

#### *Lactobacillus crispatus* viability assay

Using the method of Rohan et al. [15], *Lactobacillus crispatus* ATCC 33197 was utilized for vaginal tissue compatibility assay. Loss of viability of *Lactobacillus crispatus* was reported as vaginal product failure [15].

#### Assessment of rectal and vaginal epithelium exposed to the bigel formulation

Thirty female adult healthy treatment naïve rats weighing 160–170 g purchased commercially from Joss Rattery® breeds farm in Ibadan, Oyo State, Nigeria, were utilized in this study. The rats acclimatize in their new environment for 5 days before study commencement maintained at 29 ± 2 °C and relative humidity (40 ± 3%) in a 12-h light and dark cycle. They had access to a standard rat chow and clean drinking water ad libitum. The animals were kept in 590 × 400 × 210 mm polycarbonate cages housed in well-aerated rooms. This study followed the National Institutes of Health guide for the care and use of laboratory animals [16]. All the experiments accorded with the Institution Guidelines and were approved in writing by Health Research Ethical Committee CMUL/HREC/10/19/645. This study utilized the Animal research: reporting in vivo experiments: the ARRIVE guidelines in documenting the study [17], and the ARRIVE checklist can be found in the supplementary materials. All rats were hormonally synchronized using 2 mg/kg/body weight medroxyprogesterone acetate administered subcutaneously 5 days prior to bigel vaginal administration. The rats were randomized into seven groups (3 rats/group) and were administered daily doses of 50 μL of the bigel formulation intravaginally and rectally using a sterile stainless steel feeding needle with a ballpoint end to assess irritation. The external appearance of the vagina was observed daily and important aspects were analyzed that would involve visible signs of damage such as difficulty in inoculation, vagina contraction, spasms, redness, or burning. No anesthesia was used during this study. On day 14, rats were humanely euthanized (via exposure to carbon dioxide gas); vaginal and rectal tissues were excised and fixed in 10% formalin solution for histological analysis [18, 19]. The sections were viewed and photographed using a Leica DM 750 microscope and an ICC HD 50 camera.

### Efficacy testing

#### TZM-bl assay

This assay was performed as described by Wei et al. [20], and determination of cytotoxic concentration as well as the effective bigel dose, i.e., CC50 and ED50 respectively, of the APIs and bigel utilized was obtained and therapeutic index was calculated using GraphPad Prism.

### Statistical analysis

Values were presented as mean ± S.E.M. Unpaired *t* test was used to test statistically significant differences between the efficacy of the individual bigel formulations and control. ANOVA and Dunnett’s test were used to test statistically significant differences within the bigel formulations. A *p* value ≤ 0.05 was considered as significant.

## Results

### Physicochemical testing of bigel formulations

All the bigel formulations were white in color with an agreeable odor and good gel consistency. The viscosity was also seen to increase as the ratio of organogel to hydrogel increased. The pH of the bigel formulations ranged from 4.0 ± 0.34 to 5.2 ± 0.18 which was consistent with the healthy human vaginal pH that ranges from 3.5 to 4.5 [21]. The bigel formulations had a spreadability ranging from 45.14 to 63.11 g.cm/s which indicates a good spread. Bigel A had the best spreading properties with a low spread of time and a high spreadability. All bigel formulations showed a good spreadability value. Osmolality of the gels were within acceptable limits; BG D had the highest osmolality of 599 ± 3.2 (mOsm/kg**)** which is lower than 1000 mOsm/kg (Table 2), hence reducing the incidence of vaginal epithelial stripping. The thermocycling study showed the separation of the bigel formulations into two phases which on cooling formed the bigel but with a lower viscosity. This demonstrates the stability of the bigel formulations. Intermediate stability data showed no significant changes in the data studied over 3 months (Table 2). This further confirms the stability of the bigel formulations.

**Table 2:** Physicochemical testing of tenofovir and maraviroc bigel formulations

Bigel	Properties	Storage condition			
		25° ± 2 °C/65 ± 5%RH		40° ± 2 °C/75 ± 5%RH	
		0 month	3 months	0 month	3 months
A	Color	White	White	White	White
	Appearance	Homogenous	Homogenous	Homogenous	Homogenous
	pH	5.2 ± 0.18	5.2 ± 0.01	5.2 ± 0.18	5.2 ± 0.01
	Viscosity (cPas) 40 rpm	14560 ± 10.8	14562 ± 11.1	14600 ± 9.32	14718 ± 4.78
	Spreadability (g.cm/s)	63.11	62.0 ± 1.01	63.67	61.0 ± 3.59
	Osmolality (mOsm/kg)	450.2 ± 4.4	451 ± 3.8	450.2 ± 4.4	456 ± 2.4
B	Color	White	White	White	White
	Appearance	Homogenous	Homogenous	Homogenous	Homogenous
	pH	4.3 ± 0.03	4.9 ± 0.32	4.3 ± 0.03	4.9 ± 0.56
	Viscosity (cPas) 40 rpm	14476 ± 9.1	14477 ± 12.3	14501 ± 10.2	14670 ± 9.9
	Spreadability (g.cm/s)	56.57	58.31 ± 1.08	57.77	60.30 ± 2.01
	Osmolality (mOsm/kg)	473 ± 2.7	473 ± 2.7	473 ± 2.7	478 ± 3.1
C	Color	White	White	White	White
	Appearance	Homogenous	Homogenous	Homogenous	Homogenous
	pH	4.4 ± 0.29	4.6 ± 0.03	4.4 ± 0.29	4.6 ± 0.18
	Viscosity (cPas) 40 rpm	14179 ± 10.1	14179 ± 14.6	14229 ± 7.3	14296 ± 11.5
	Spreadability (g.cm/s)	45.14	48.7 ± 2.01	46.94	49.1 ± 1.99
	Osmolality (mOsm/kg)	430 ± 4.9	431 ± 2.1	430 ± 4.9	439 ± 1.27
D	Color	White	White	White	White
	Appearance	Homogenous	Homogenous	Homogenous	Homogenous
	pH	4.0 ± 0.34	4.9 ± 0.01	4.0 ± 0.34	4.9 ± 0.06
	Viscosity (cPas) 40 rpm	14293 ± 7.9	14299 ± 9.2	14371 ± 8.1	14498 ± 12.8
	Spreadability (g.cm/s)	47.44	45.33 ± 1.99	47.99	46.01 ± 1.29
	Osmolality (mOsm/kg)	590 ± 7.4	599 ± 3.2	590 ± 7.4	603 ± 1.93

### Microscopic analysis

**Fig. 1:**
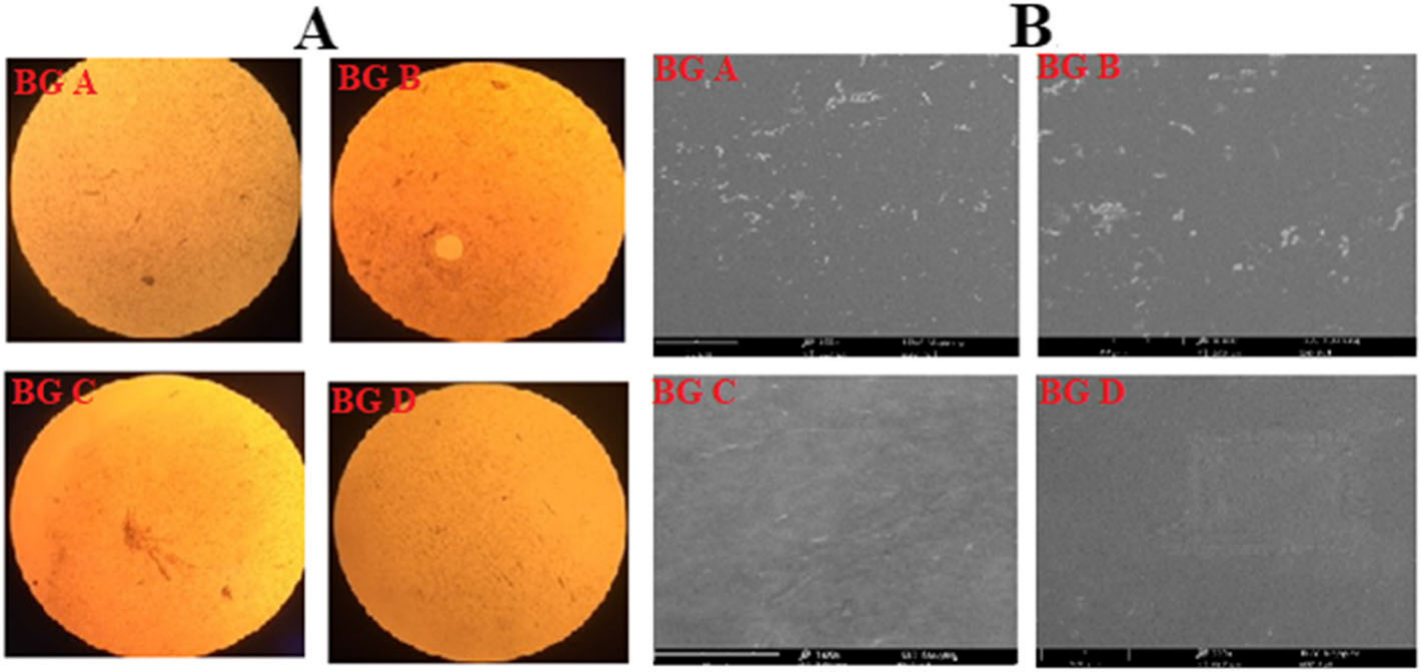
**a** Light microscope images of the maraviroc/tenofovir bigel formulations BG A, BG B, BG C, and BG D. **b** SEM image of air-dried maraviroc/tenofovir bigel formulations BG A, BG B, BG C, and BG D

Microscopic examination of the gels using a light and scanning electron microscope showed the gel to possess fiber-like structures because of entrapment of the organogel in the hydrogel molecules as seen in Fig. 1. This entrapment was seen to be uniformly achieved hence the stability of the formulations. Uniform structural properties of bigel shown by SEM are due to the better homogenization of the gels (Fig. 1b).

### Compatibility studies

**Fig. 2:**
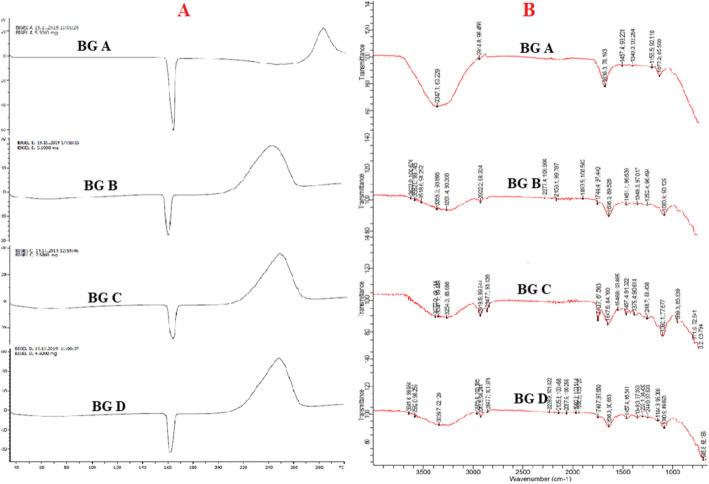
**a** DSC scans of maraviroc/tenofovir bigel formulations BG A, BG B, BG C, and BG D. **b** FTIR spectra of maraviroc/tenofovir bigel formulations BG A, BG B, BG C, and BG D

The FTIR spectra of the bigel formulations show the presence of absorption peaks corresponding to the bigel formulations without the drug as seen in Fig. 2b. N–H bending band at 1622 cm^−1^ with expansion 400–2500 cm^−1^ along with stretching vibration of –NH_2_ and –OH groups are observed at 3200–3500 cm^−1^ as shown in Fig. 2b characteristic of tenofovir in the spectra. Tenofovir presence was also detected via the presence of vibration at 1674 cm^−1^, 1255 cm^−1^, and 1751 cm^−1^ indicative of imine, primary aromatic imines, and carbonyl groups respectively. Maraviroc exhibited a broad absorption band at 2934 cm^−1^, due to stretching vibration of the amide N–H group, a sharp band at 1663 cm^−1^ due to the carbonyl group, and 1530 cm^−1^ band due to amide N–H group. An increase in intensity of the peak was observed from bigel BG A to BG D; this indicated an increase in the hydrogen bonding among the gel components [22]. This indicated that intermolecular hydrogen bonding played a major role in the formulation of bigel. No extra peak was observed in the bigel formulations with the drug as compared to bigel without drug which shows that the presence of tenofovir and maraviroc did not cause any change in the FTIR spectra. The DSC thermogram as seen in Fig. 2a shows the presence of an exothermic crystallization peak, endothermic peak, and a *T*_g_ (glass transition temperature) at 40 °C. The endothermic peak indicates the presence of water which vaporizes on heating while the exothermic peak indicates a rearrangement of molecules to form crystals. The results indicate that the bigel formed is thermoplastic, and it is present in a semi-crystalline state. No incompatibility was detected.

### Mucoadhesion study

Mucus penetrating of formulations utilized in vaginal drug delivery is a critical retention of formulation on the vaginal mucosal surface [11]. The percentage mucoadhesion obtained for the formulations was 53.87% ± 2.11, 55.78% ± 3.91, 67.99% ± 3.77, and 60.17% ± 1.01 respectively for maraviroc/tenofovir bigel formulations BG A, BG B, BG C, and BG D. Maraviroc/tenofovir bigel formulation BG C had the highest mucin adsorption followed by BG D. For the gels to be maximally bioavailable, they should have a mucin adsorption higher than 40% [11, 22]; this was seen in all the formulations developed.

### In vitro drug release rate

**Fig. 3:**
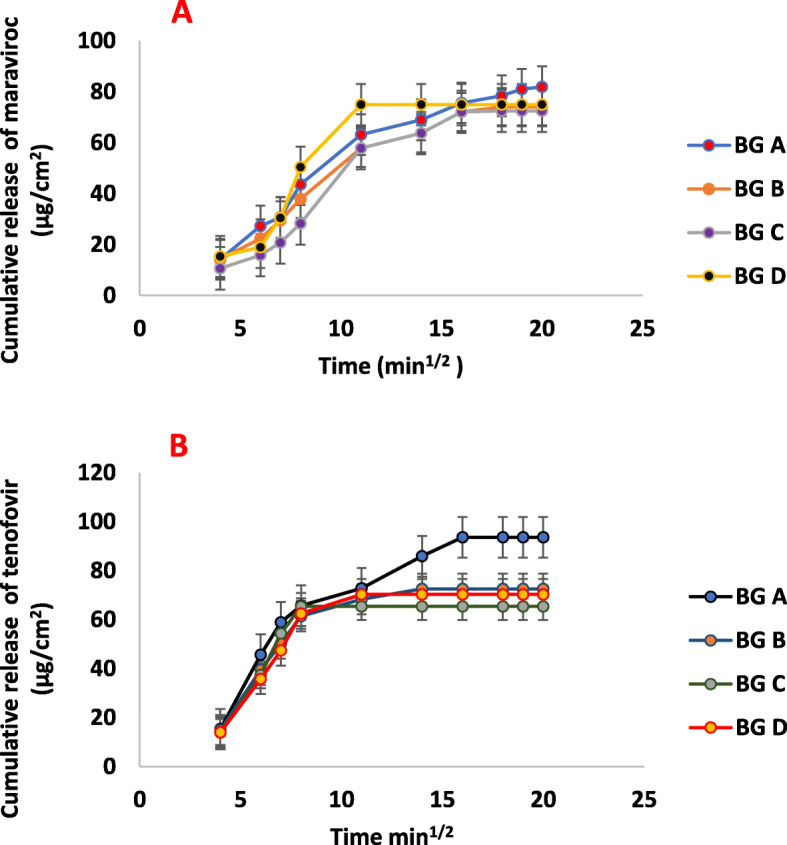
**a** Cumulative release of maraviroc from bigel formulations. **b** Cumulative release of tenofovir from bigel formulations

The in vitro release study of maraviroc from the bigel formulations showed a release rate ranging from 2.675 to 3.838 μg/cm^2^/min^½^ while the release rate for tenofovir ranged from 3.475 to 3.825 μg/cm^2^/min^½^. The release as seen from Fig. 3a, followed a zero-order release for maraviroc. When the values were fit into a zero-order mathematical equation, *k*_o_ was constant and the *r*^2^ was approximately 0.938. This mechanism indicates that the release is independent of concentration and that release of maraviroc occurs through diffusion across the bigel. The mechanism of release of tenofovir as seen in Table 3 and Fig. 3b fits into the Korsmeyer-Peppas equation as the *k* values obtained when values were fit into the Korsmeyer-Peppas equation were constant. Using one sample *t* test, comparing the mean of the release of the drugs in each of the bigel with a hypothetical mean of 100 showed that BG C and BG D had the most statistically significant release of tenofovir while BG B and BG C had the most significant release for maraviroc.

**Table 3:** In vitro drug release kinetic study of the API from the various tenofovir and maraviroc bigel formulations

In vitro *drug release kinetic* study for maraviroc								
	**Zero order**		**First order**		**Higuchi**		**Korsmeyer-Peppas**	
**Formulation**	***r***^**2**^	***k***_**0**_	***r***^**2**^	***k***_**1**_	***r***^**2**^	***k***_**2**_	***r***^**2**^	***k***_**3**_
BG A	0.938	4.176	0.8323	0.0928	0.9702	28.22	0.9473	4.117
BG B	0.9355	3.8941	0.8529	0.0931	0.9662	26.30	0.9575	3.825
BG C	0.9179	4.309	0.8604	0.1166	0.9439	29.04	0.9502	1.834
BG D	0.776	3.833	0.7253	0.0912	0.8343	26.39	0.8538	4.077
In vitro *drug release kinetic* study for tenofovir								
	**Zero order**		**First order**		**Higuchi**		**Korsmeyer-Peppas**	
**Formulation**	***r***^**2**^	***k***_**0**_	***r***^**2**^	***k***_**1**_	**Formulation**	***r***^**2**^	***k***_**0**_	***r***^**2**^
BG A	0.8412	4.0938	0.6362	0.0764	0.9000	28.14	0.8159	4.89
BG B	0.6979	2.7713	0.5532	0.0636	0.7781	19.45	0.7875	5.40
BG C	0.5171	2.1370	0.4575	0.056	0.6020	15.32	0.6882	5.84
BG D	0.6552	2.7074	0.5404	0.0653	0.7374	19.08	0.7859	4.74

### Safety testing

**Fig. 4:**
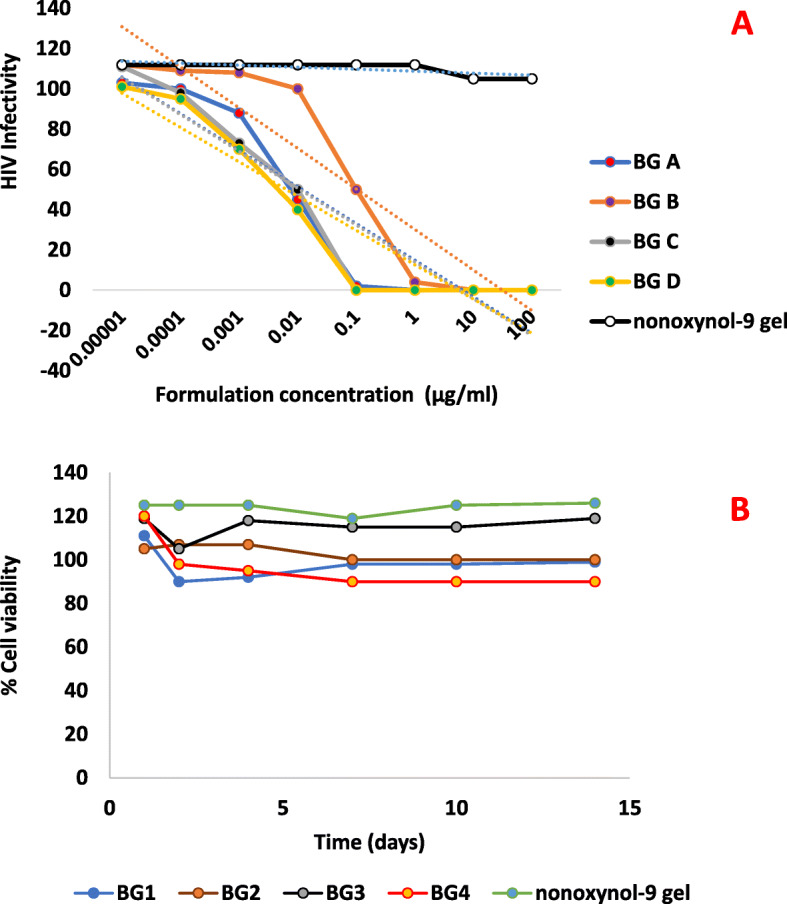
**a** HeLa cell cytotoxicity of API from maraviroc tenofovir bigel formulations; results are expressed as % cell viability compared to HeLa cells without any treatment (*n* = 3 ± SD). **b** HIV infectivity dose-response curves for maraviroc from maraviroc tenofovir bigel formulations incubated with HIV-1 indicator TZM-bl cells at different concentrations (*n* = 5 ± SD)

**Fig. 5:**
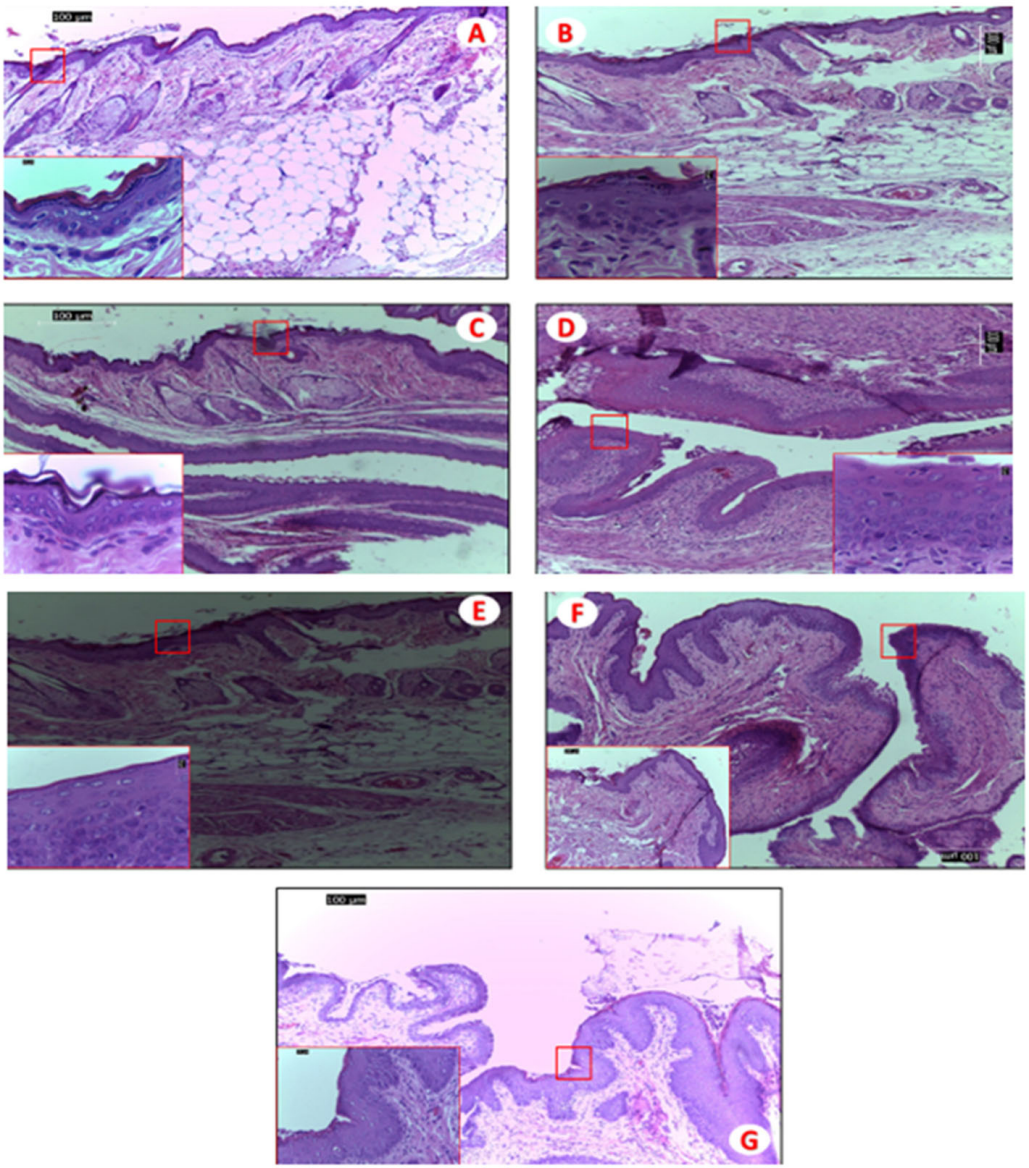
Hematoxylin and eosin-stained sections of mice vagina. Light micrographs (× 100 and × 1000 (red square) of the vaginal mucosa of mice after 14 days of daily treatment with 0.04 mls of 1:20 dilution. **a** BG A. **b** BG B. **c** BG C. **d** BG D. **e** Bigel without maraviroc and tenofovir. **f** N9 gel. **g** No treatment. Photographs are representative of all treated mice (*n* = 3 group). Original magnification × 1000

**Fig. 6:**
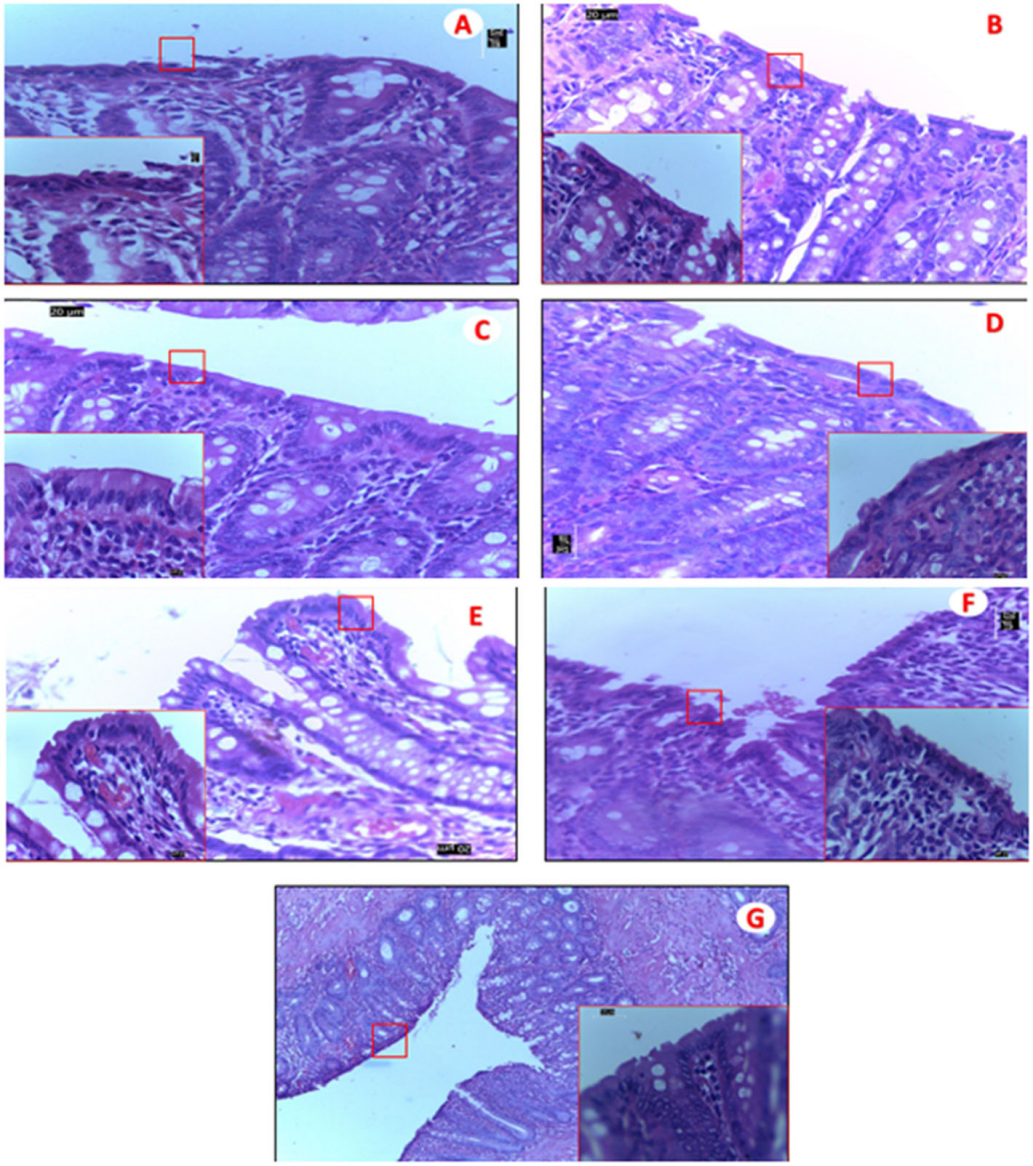
Hematoxylin and eosin-stained sections of mice rectum. Light micrographs (× 400 and × 1000 (red square) of the rectal mucosa of mice after 14 days of daily treatment with 0.04 mls of 1:20 dilution. **a** Bigel A. **b** Bigel B. **c** Bigel C. **d** Bigel D. **e** Bigel without drugs. **f** N9 gel. **g** No treatment. Photographs are representative of all treated mice (*n* = 3 group). Original magnification × 400

HeLa cell lines which are representative of female genital tract were exposed to a 1:10 dilution of the gel. BG B, BG D, and BG A showed a loss of viability below 100 % but not less than 90% loss. The loss of viability for BG D was the most which also appeared so in the safety test for normal vagina flora. BG C showed a viability like the control cells (Fig. 4b). The results showed a reduction in the number of colonies which were within acceptable limits (Fig. 4b; Table 4). All the bigel formulations except for BG D showed no irritation or inflammation in the vaginal epithelia of the rats studied as compared with the vagina epithelium of the control (no treatment group) (Fig. 5). The stratified squamous epithelium was observed to be intact. BG D caused a distortion of epithelium with slight hyperplasia coupled with the greatest loss of viability in the test against normal vagina flora (Fig. 5d). The rectal tissue exhibited intact simple columnar epithelium except for the BG A and D that showed distortions in the epithelial lining having more of squamous cells rather than simple columnar epithelium (Fig. 6a-d).

**Table 4:** *Lactobacilli crispatus* viability testing of tenofovir and maraviroc bigel formulations

Formulations	Untreated growth yield (cfu)	Treated growth yield (cfu)	Log reduction (official limit not ≥ 1)
BG A	50	48	0.07
BG B	50	46	0.036
BG C	50	36	0.142
BG D	50	35	0.155

### Efficacy testing

All the bigel formulations showed a decrease in HIV infectivity at a concentration of 0.00001 μg/mL using TZM-bl cells. There was a continuous decline in HIV infectivity until at a concentration of 0.1 μg/mL where there was no infectivity except for bigel B which achieved a zero level of infectivity at a concentration of 1 μg/mL (Fig. 4a).

## Discussion

Sexual transmission of HIV is the most common means of acquiring the disease [1]. Attempts have been made to provide drugs and methods to prevent, treat, and manage the transmission of HIV infection through sexual interactions [23]. Some of the most novel efforts in preventing HIV transmission include the use of microbicides [24, 25]. Several non-specific microbicides have been developed and advances made with ARV-based microbicides. These classes of drugs have however only gained limited popularity due to the emergence of drug resistance strains, chemical and enzymatic instability, and poor adherence [26]. This study focused on the enhancement of the efficacy of tenofovir 1% gel by combining tenofovir and maraviroc as a bigel while taking into consideration the hydrophilicity of tenofovir and the hydrophobicity of maraviroc. The gels were of good esthetics that could enhance patient’s adherence. The choice of hyaluronic acid as the base for the hydrogel was to limit irritation and toxicity by simulating the natural constituent of the vaginal fluid. Palm oil was used as the base of the organogel because of its natural source, affordability, and accessibility. These factors will subsequently have positive effects in the cost of production and thus affordability of the product when the research findings are translated into products.

Consumer preference and adherence for a product depends on various properties of the preparation, which includes appearance, odor, initial sensations upon contact with the area applied, spreading properties, and greasiness [27, 28]. The bigel formulations appeared white and had a slight characteristic smell of the oily phase. They had a good feel on the skin and exhibited a pseudoplastic behavior as their viscosity was seen to reduce with increased temperature. This property ensured that the developed bigel formulations had adequate bio adhesion and facilitated drug release [29]. This property will promote the retention of the drug in the gel until it gets into the vaginal mucosa where the slight difference in temperature will modulate its viscosity and enhance mixing with the vaginal fluid for optimum efficacy. The viscosity was also seen to increase as the ratio of organogel to hydrogel increased. Spreadability of a gel gives insight into the extrudability of the gel, ease of application, even distribution of dosage around the area, and ultimately patient’s acceptability [30]. The gels showed excellent spreadability hence the increased availability of maraviroc on the vaginal wall after application. This will ensure that maraviroc is present in the vagina before HIV 1 exposure via coitus.

Rapid drug release was observed from the bigel formulations as significant drug concentrations were detected at 120 s into the release study. This is particularly important for the ability of the bigel to block viral entry. The in vitro release study of maraviroc from the bigel formulations showed a release rate ranging from 2.675 to 3.838 μg/cm^2^/min^½^ while the release rate for tenofovir ranged from 3.475 to 3.825 μg/cm^2^/min^½^.

A zero level of infectivity at a concentration of 1 μg/mL was obtained for all the bigel formulations. The results of the statistical analysis showed a significant *p* values for the four bigel formulations compared to N9 gel using an unpaired *t* test with Welch correction. BG D showed the best anti-HIV activity with a *p* value of 0.0026 and *r*^2^ of 0.74. BG C was next with a *p* value of 0.004. ANOVA test using Dunnett’s multiple comparison also showed BG D to have the best anti-HIV activity as compared to nonoxynol 9 gel. BG D showed no statistically significant difference in activity when compared with the control, nonoxynol 9 gel.

The presence of *Lactobacillus* species and other bacterial species in a healthy human vagina contributes to maintain the 3.5 to 4.5 acidic pH and produces several antiviral and antimicrobial substances that inhibit pathogenic organisms [31]. Alterations of normal vaginal microflora may lead to several vaginal infections and affect the risk for vaginal HIV transmission. Drugs formulated for administration into the vagina must thus not alter the normal microflora of the vagina. BG A had the lowest reduction in the number of colonies while BG B had the highest reduction. The results obtained indicate that when applied vaginally, the bigel formulations are expected to maintain the level of microflora within acceptable limits and no irritation or harmful effects are expected.

Histopathology studies of the rectal tissues of rats showed inflammation and alteration in the rectal epithelium of rats administered BG A and D, although BG A showed no loss of vaginal epithelium. However, a loss of vagina epithelium was observed in BG D. While these results rules out the potential of BG D as a microbicide for both rectal and vagina use, it shows the potential of BG A as a vaginally administered microbicide. BG B showed slight inflammation of the epithelium which might have been as a result of the administration process rather than toxicity of the gel. Rats administered BG C, positive and negative control and no treatment group, maintained integrity of the rectal epithelium. BG C shows safety against vaginal and rectal epithelium which suggests its use as a potential dual compartment bigel to prevent HIV transmission. These data are in contrast with the results of the study of Hladik et al. [32] which reported the potential toxic effect on the rectal and vagina tissues associated with the use of tenofovir gel, hence the need to develop dual compartment formulations which are in tandem with the solubility profiles of the active pharmaceutical ingredients. Dual entity bigel containing maraviroc and tenofovir are useful especially where resistance to non-nucleoside transcriptase inhibitor NNRTIs is present.

## Conclusion

To achieve the UNAIDS 95-95-95 goal, a major focus on intervention must be holistically taken into consideration to include pre- and post-exposure prophylaxis medications, PMTCT among others as well as microbicide use. This study successfully developed a dual compartment bigel containing maraviroc and tenofovir. BG C was found to be stable and safe towards vaginal and rectal epithelium, and it actively prevented HIV transmission. This bigel has the potential for long-term pre-exposure prophylaxis prevention of HIV transmission.

## Supplementary information

**Additional file 1.** The ARRIVE Guidelines Checklist

## Data Availability

All data and materials are available upon request.

## Abbreviations

ARV: Antiretroviral
HIV: Human immunodeficiency virus
NRTI: Nucleotide reverse transcriptase inhibitor
BG: Bigel
PBS: Phosphate-buffered saline
