# Supplementary material for: Development and evaluation of mucoadhesive bigel containing tenofovir and maraviroc for HIV prophylaxis
Source: Futur J Pharm Sci. 2020 Nov 14;6(1):81. doi: 10.1186/s43094-020-00093-3 (PMC7678373; doi:10.1186/s43094-020-00093-3)

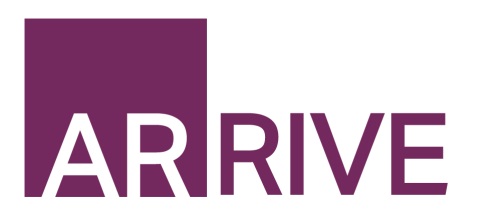


The ARRIVE Guidelines Checklist for research titled “Development and evaluation of mucoadhesive bigel containing tenofovir and maraviroc for HIV prophylaxis”

Animal Research: Reporting In Vivo Experiments

Carol Kilkenny^1^, William J Browne^2^, Innes C Cuthill^3^, Michael Emerson^4^ and Douglas G Altman^5^

*^1^The National Centre for the Replacement, Refinement and Reduction of Animals in Research, London, UK, ^2^School of Veterinary Science, University of Bristol, Bristol, UK, ^3^School of Biological Sciences, University of Bristol, Bristol, UK, ^4^National Heart and Lung Institute, Imperial College London, UK, ^5^Centre for Statistics in Medicine, University of Oxford, Oxford, UK.*

|  | | ITEM | RECOMMENDATION | Section/ Paragraph |
| --- | --- | --- | --- | --- |
| 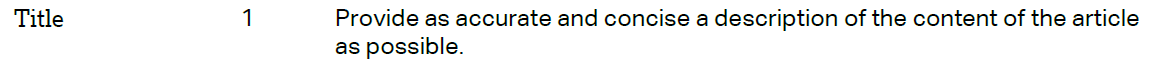 | | | TITLE |  |
| 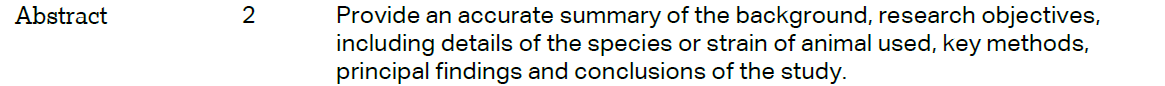 | | | ABSTARCT |  |
| INTRODUCTION | | |  |  |
| 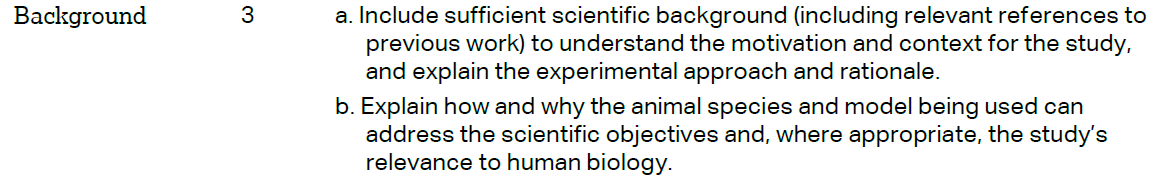 | | | Paragraph 1 and 2 |  |
| 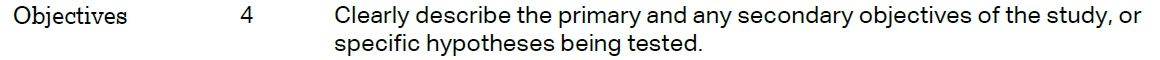 | | | Paragraph 3 |  |
| METHODS | | |  |  |
| 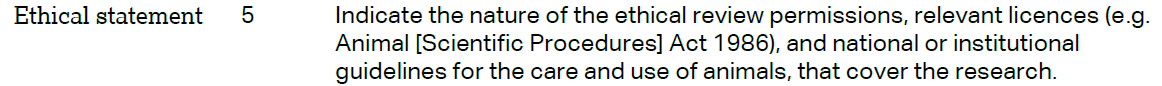 | | | Page 7 |  |
| 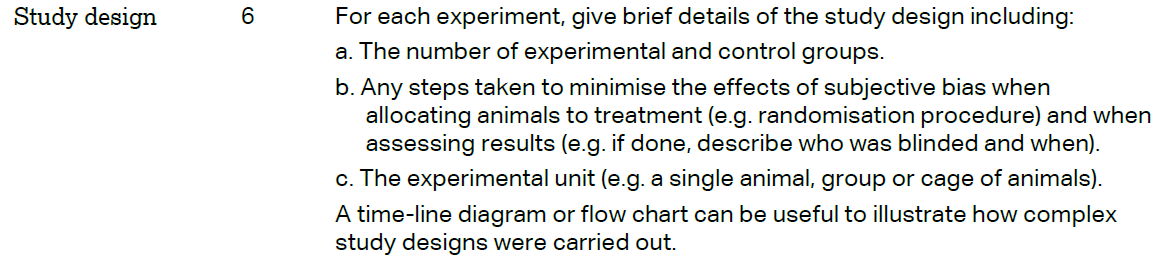 | | | Page 7 |  |
| 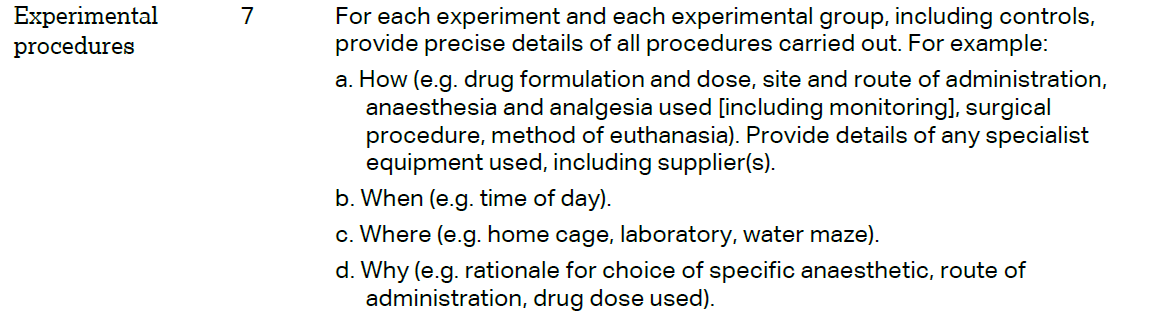 | | | Page 7 |  |
| 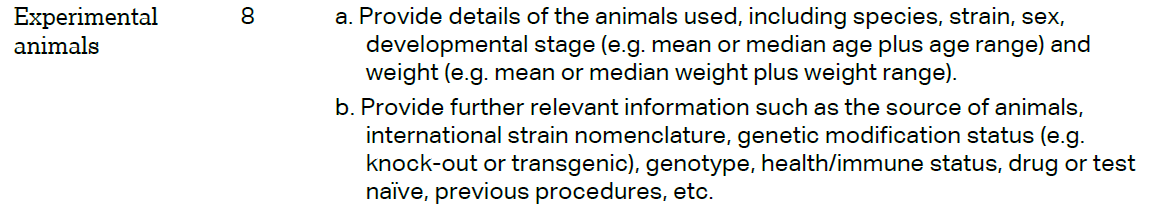 | | | Page 7 |  |

The ARRIVE guidelines. Originally published in *PLoS Biology*, June 2010^1^

| 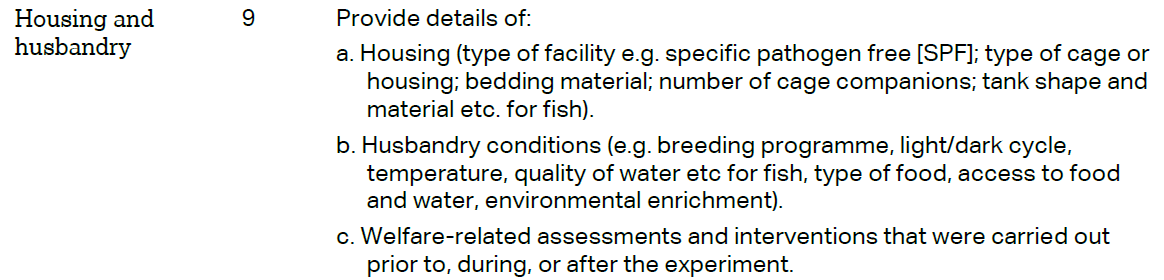 | Page 7 |  |
| --- | --- | --- |
| 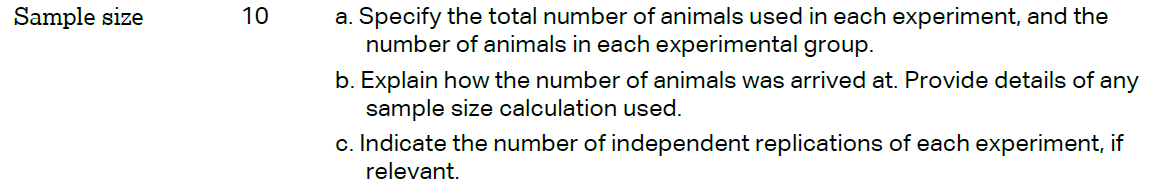 | Page 7 |  |
| 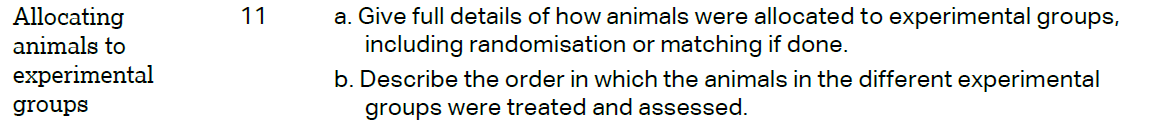 | Page 7 |  |
| 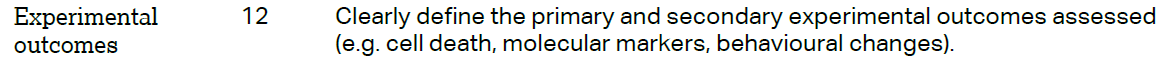 | Page 7 |  |
| 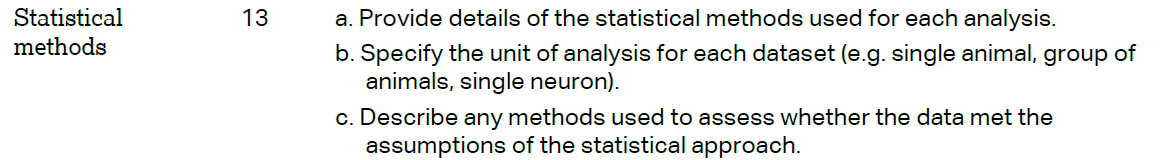 | Page 7 |  |
| RESULTS |  |  |
| 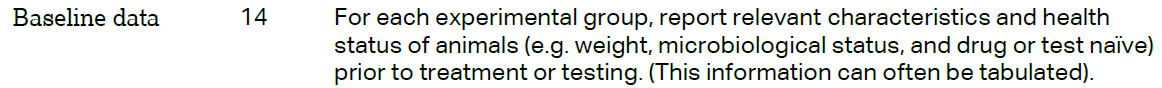 | Page 11-12 |  |
| 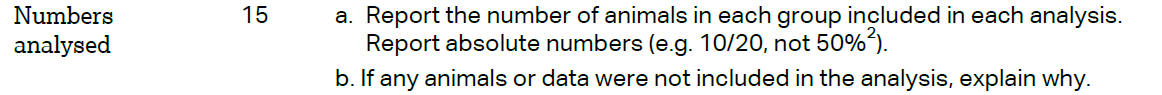 | Page 11-12 |  |
| 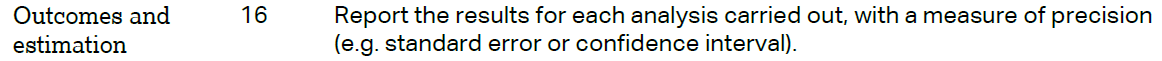 | Page 11-12 |  |
| 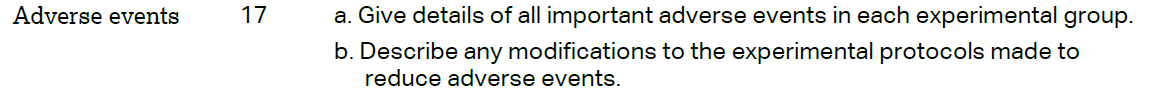 | Page 11-12 |  |
| DISCUSSION |  |  |
| 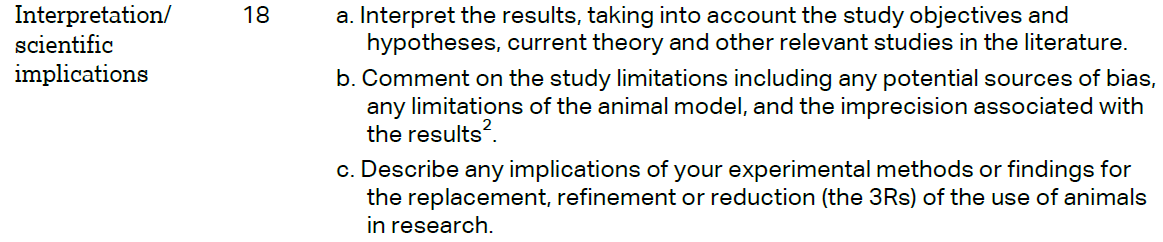 | Discussion section |  |
| 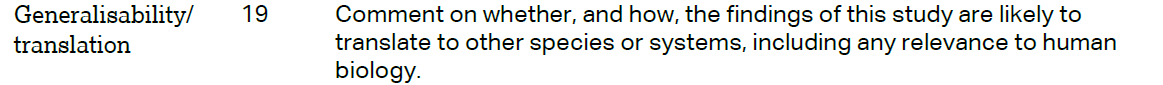 | Discussion |  |
| 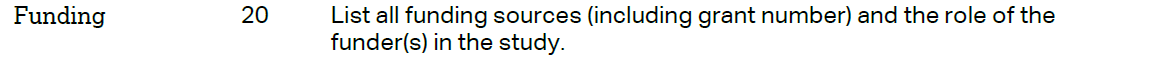 | | Listed |


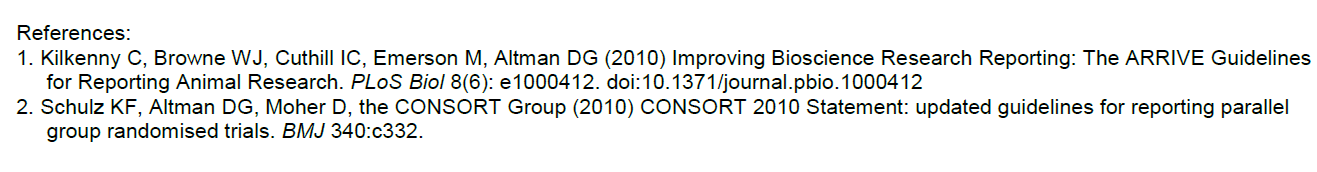

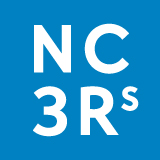

Supplement: Supplementary file 1 — Additional file 1. The ARRIVE Guidelines Checklist [file 43094_2020_93_MOESM1_ESM.docx]
